# Eliciting candidate anatomical routes for protein interactions: a scenario from endocrine physiology

**DOI:** 10.1186/1471-2105-14-131

**Published:** 2013-04-16

**Authors:** Pierre Grenon, Bernard de Bono

**Affiliations:** 1 EMBL‐EBI, Wellcome Trust Genome Campus, Hinxton, Cambridgeshire, CB10 1SD, UK; 2 ABI, University of Auckland, Symonds Street, Auckland 1010, New Zealand; 3 CHIME Institute, Archway Campus, University College London, London UK

## Abstract

**Background:**

In this paper, we use: i) formalised anatomical knowledge of connectivity between body structures and ii) a formal theory of physiological transport between fluid compartments in order to define and make explicit the routes followed by proteins to a site of interaction. The underlying processes are the objects of mathematical models of physiology and, therefore, the motivation for the approach can be understood as using knowledge representation and reasoning methods to propose concrete candidate routes corresponding to correlations between variables in mathematical models of physiology. In so doing, the approach projects physiology models onto a representation of the anatomical and physiological reality which underpins them.

**Results:**

The paper presents a method based on knowledge representation and reasoning for eliciting physiological communication routes. In doing so, the paper presents the core knowledge representation and algorithms using it in the application of the method. These are illustrated through the description of a prototype implementation and the treatment of a simple endocrine scenario whereby a candidate route of communication between ANP and its receptors on the external membrane of smooth muscle cells in renal arterioles is elicited. The potential of further development of the approach is illustrated through the informal discussion of a more complex scenario.

**Conclusions:**

The work presented in this paper supports research in intercellular communication by enabling knowledge‐based inference on physiologically‐related biomedical data and models.

## Background

A considerable proportion of physiological, pharmacological and disease processes involves the interaction between proteins (i.e. peptides, polypeptides, or their complexes) across distinct subcellular, tissue and anatomical compartments. In particular, such protein interactions play a key role in mediating communication between cells that participate in juxtacrine (e.g. Notch signaling [1]), paracrine (e.g. IGF‐1 [2]), endocrine (e.g. thyroid hormone action [3]) and exocrine (e.g. immunological factors passed on via lactation [4]) processes.

Direct protein interactions are realised by processes involving two or more proteins that bind directly with one another. Two key prerequistes for such an occurrence are that: 1) interacting proteins are spatially co‐located in the same portion of a compartment, and 2) the molecular constituents of that site are well mixed. For non co‐located cells to communicate, therefore, at least one of the interacting proteins produced by one of the cells must translocate to the site of location of its binding partner produced by the other cell. This requirement for translocation mechanisms is fulfilled by physiological processes that include transport modalities such as diffusion, advection and convection. Such mechanisms take place along a route, in a succession of sites, via a series of these distinct physiological transport modalities.

A simple example of a short communication route is that taken by a protein diffusing from the bloodstream in the capillaries of a given organ (e.g. coronary microcirculation) to the extracellular tissue fluid compartment of that organ (e.g. tissue fluid in the left ventricular wall). In this case, the anatomical route starts inside a capillary and ends in the extracellular tissue fluid with an intermediate step in the endothelial intercellular space during the crossing of the capillary wall by the protein as it is filtrated by the capillary. This example is simplified, of course, because in general the capillaries are not the production sites of their filtrate. Moreover, even such a simple example shows the potential complexity of giving an account of such phenomena in that: 1) sites (i.e. portions of blood in the capillaries of an organ, as well as portions of tissue fluid of that same organ) need to be identified and 2) transport modalities need to be taken into account relative to the translocating objects and their physical and chemical characteristics.

The latter point is crucial for the representation of communication processes involving translocation mechanisms, since prevailing biophysical conditions may facilitate or impede particular mechanisms. For example, vascular insufficiency may reduce the rate of translocation between capillaries and tissue fluids connected to each other. Conversely, a state of inflammation may increase the rate of translocation as the overall endothelial intercellular gap space is increased. These examples draw attention to the complexity of barrier crossing mechanisms and the regulation of accessibility between regions for different kinds of translocated proteins. These aspects are pervasive, multiscale and arise in greater number as routes of communication become more complex.

It is not straightforward to find anatomical translocation routes for specific pairs of interacting proteins—or for the translocation of any other kind of small molecule. Protein‐protein interactions data—as well as a wealth of data of varied sorts—can be found in a number of databases which are maintained at great expense by the biomedical community. These databases can be very large and also very specialised. Naturally, they do not contain all relevant anatomical or physiological knowledge. Increasingly, however, these databases include controlled vocabulary and pointers to ontologies making them potentially connected to knowledge representations in their domains; such pointers are, sometimes, to relevant anatomical locations. There is no shortage of anatomical knowledge and yet it is not always readily available in database format or not always to a realistic degree of detail and completion. Furthermore, should such data become available, the finding of translocation routes would remain a challenge; indeed, the task would involve processing data and inferring implicit facts.

Knowledge representation and reasoning (see [5] for a short overview and [6] for a more thorough treatment) become relevant to bridge the gaps described in the foregoing and to combine and articulate data in distinct, specialised biological domains. This paper demonstrates how the combination of data (in the form of ground facts about proteins and their interactions, on the one hand, and body compartment connectivity, on the other hand) and knowledge (in the form of a formal theory of physiological communication between body compartments) can be achieved using knowledge representation and reasoning techniques. The paper describes a reusable and reimplementable elaboration of a knowledge base in the logic‐based knowledge representation tradition (see [7]). This elaboration results in an expert system for answering physiological connectivity queries on top of a knowledge base of anatomical connections and in combination with knowledge about translocating objects (here, proteins).

A direct contribution of the paper is to specify (parts of) a formal theory of physiological communication across anatomical compartments that may be used to query biomedical data. A method is contributed too, albeit through a specific illustrative case study, and points toward the construction and maintenance of tools and resources for using biomedical data (here, protein interaction data). The work is, however, prototypical and the achievement of code release and resource deployment still requires further development. An indirect contribution of the paper is the exemplification provided by the discussion of how a knowledge base system can be used in connection to other pieces of software (e.g. fast implementations of graph traversal algorithms) so as to make use of data curated in biomedical databases.

The paper presents knowledge representation requirements for the elicitation of routes of communication addressing the above complexity and the core of a theory addressing these requirements. To this end, the paper uses a scenario from endocrine physiology of manageable complexity for the purpose of discussion. In this scenario, a protein hormone is secreted by a cell and released into the bloodstream. This hormone then reaches an anatomically distinct site where it binds to its receptor deployed at the surface of another cell (in another tissue). The present treatment is primarily concerned with translocation pathways for molecules at a physiological level. This scenario assumes a process of protein synthesis that ends with the placement of the final product in certain subcellular sites defining the boundaries of communication routes. Furthermore, each protein is assumed to be located in one or more of three partitioning subcellular compartments, namely: i) cytoplasm, ii) plasma membrane, iii) extracellular space. Our purpose is to identify ways in which sites in these compartments may be linked to allow translocation processes to occur—by extension our approach applies to other substances than hormones.

The specific endocrine process studied in this work is the translocation of Atrial Natriuretic Peptide (ANP) hormone from the wall of the cardiac atria to the extracellular tissue fluid in kidneys, where ANP binds to the cell‐surface receptor ANPr. The motivation for our choice is three‐fold: 1) experimental research on ANP endocrinology is well established [8], 2) this endocrine process is linked to a number of common disease scenarios (e.g. [9]‐[11]), and 3) mathematical modelling of this process (e.g. as part of the Guyton model of circulation [12]) provides a quantitative framework explicitly relating rates of hormone secretion with the cardiovascular effect on the kidneys.

The ANP work in this paper is illustrative of the proposed method which consists in logically defining and constructing anatomical paths as ordered series of segments in a graph capturing transport modalities between sites in the body. In the context of the above endocrine scenario, the method and the theory presented are applied to a knowledge base of human anatomical connectivity statements in order to elicit candidate routes that link the cardiac location of ANP production to the renal site of ANPr.

The next section presents the knowledge representation requirements and a theory which addresses them. The section after the next presents a prototype system in the context of the ANP use case. This is followed by a discussion section before finishing the paper with a section containing conclusive remarks.

## Method

The knowledge representation requirements for the task of eliciting communication routes for protein interactions can be summarised as follow: 

1. Representation of ground facts, which requires the ability to: 

(a) represent proteins as spatial entities,

(a) represent protein interactions as spatial processes,

(a) represent tissue and subcellular localisations of proteins—for production sites as well as for interaction sites;

2. Triage: Ability to select the kind of interaction and the kind of candidate routes based on heuristics and a knowledge base, which includes the ability to: 

(a) represent kinds of physiological communication,

(a) ascribe to proteins their capability to follow a route of a given kind,

(a) carry inference—for the classification of an interaction and the selection of routes and fragments thereof—based on necessary and sufficient conditions for the occurrence of a given interaction;

3. Route identification and output representation, which requires the ability to: 

(a) represent routes of communication as entities in their own right,

(a) represent compartments spanned by routes as discrete body portions,

(a) represent complex routes involving distinct connectivity systems in the body,

(a) carry inference for the elicitation of routes with a declarative knowledge representation theory supporting procedural implementations of algorithms for route construction.

The basis for fulfilling knowledge representation and reasoning requirements of the above sort consists of: i) a vocabulary to express ground facts and those facts that ought to be derivable through inferencing and ii) a theory axiomatising the vocabulary and informing the inferences using it. Together, these constitute an ontology for the targetted domain knowledge. In our case, however, the resulting ontology combines elements of different levels of generality in a broad range of domains. We proceed from general to specific and provide elements of axiomatisation supporting inferences. The presentation leads progressively to an algorithm integrating the surveyed representation and governing the elicitation of a route of communication for the interaction of proteins. Our formalisation uses first‐order predicate logic: → stands for material implication (read ‘implies’ or ‘if …then …’), ∧ for conjunction (read ‘and’) and ∃ for ‘there exists’. Propositions are numbered in the order of their appearance and unbound variables are assumed to be universally quantified. For convenience, symbols for relations use lower‐case strings and symbols for categories and individuals start with an upper‐case. For further convenience, each proposition is followed by a sentence proposing a literal way of reading it and a paraphrase. For ease of reference, we collect the knowledge representation elements used in the present formalisation below.

### Knowledge representation at a glance

Many of the elements listed have been given varied treatments, especially those in the spatial domain; they are introduced and explained in what follows to the extent required for our purpose.

#### Categories and individuals

For categories and individuals, symbols used in propositions numbered in the text appear in parentheses.

Substantial (Subst) 

 Tissue 

  Cardiovascular tissue (CVS)

 Tissue structure and portion (TS) 

  Plasma‐membrane (PM)

 Fluid portion (FP) 

  Tissue Fluid (TF)

  Blood plasma

 Biological agent 

  Protein

 Location types: Ltcyt, Ltpm, Ltec

Processual (Proc) 

 Interaction

 Production

 Translocation 

  Diffusion (Dif)

  Advection (Adv)

  Convection (Cnv)

#### Relations

Table 1 lists relations in alphabetical order of their symbols. The types of arguments taken by relations appear in square brackets and a natural language reading is provided for each.

**Table 1:** Relations used in the present formalisation

**Symbol**	**Arguments**	**Reading**
acc	[Subst, Subst]	Can access
att‐to	[Subst, Subst]	Is attached to
cap‐of	[CVS,CVS]	Capillary of
CIVSR	[Subst, Subst]	Can interact with,
		via some route
CMTP_*t*_	[Subst, one of:	Can move through
	Dif, Adv, Cnv]	path type
CONN	[Subst, Subst]	Is connected to
COMM3	[Subst, Subst, one of:	Type of communication
	Dif, Adv, Cnv]	between
exp‐to	[Subst, Subst]	Is exposed to
fnd‐in	[Subst, Subst]	Is found in
fnd‐in‐postp	[Subst, Subst]	Is found in,
		post‐production
from‐loc	[Translocation, Subst]	From location
int‐btw	[Subst, Subst,	Interaction between
	Interaction]	
occurs‐in	[Proc, Subst]	Occurs in
participates in	[Subst, Proc]	Participates in
postp_*t*_	[Subst, one of:	Type of post‐production
	Ltcyt, Ltpm, Ltec]	site
prod‐by	[Subst, Subst]	Produced by
pm‐of	[PM, Subst]	Plasma membrane of
tf‐of	[FP, Subst]	Tissue fluid of
to‐loc	[Translocation, Subst]	To location
trans‐subtends‐int	[Translocation,	(translocation) subtends
	Interaction]	(interaction)
tr‐obj	[Translocation, Subst]	Translocation’s object
ts‐of	[TS, Tissue]	Tissue structure of

### General ontology and anatomical scope

Our ontological treatment involves two main categories of entity: i) spatial or material objects and ii) processes. Furthermore, these categories are associated with a number of general relations. We assume a treatment of these general categories and relations along the lines of the Basic Formal Ontology (BFO) [13]. Thus, biological objects, structures and parts of the body fall under the BFO kind *Substantial* and the kinds of process we consider fall under the BFO kind *Processual*. While we assume relevant formal treatments of these high level categories, our present formal treatment only uses the relation *participates‐in* to link substantials to a process in which they participate. Also, we use vocabulary derived from the BFO primitive relations of location for substantial entities (*located‐in* and *contained‐in*) and processual entities (*occurs‐in*). While primitive relations of location tie entities to their exact location, we allow a standard generalisation whereby location relations also link to sites containing exact locations as parts [14]. The resulting apparatus allows us to regard body compartments as sites of location of substantial and of occurrence of processes.

On the side of substantials, we require reference to various biological structures and the roles they play in different contexts. There are two main kinds: i) proteins which, in the present context, are the primary participants in interactions and translocations and ii) tissues and organs, as well as various fluid subcompartments thereof, in which translocations of proteins can occur. We declare protein types rather than actual protein instances.

On the side of processes, we are primarily concerned with processes that characterise routes of translocations leading to interactions. Thus, we are concerned with: i) protein production processes as they define potential boundary locations for translocations, ii) protein interactions as they define interaction pairs and, by way of consequence, reference‐ and target‐locations for an interaction, and iii) translocation processes within certain body structures as they define the routes of translocation. We declare process types rather than actual process instances and we do not apply a temporal treatment in the present account.

Protein interaction processes are spatial processes and can only occur in sites where interacting proteins are exposed one to the other. Exposure, *exp‐to*, is a general relation which holds when a substantial is exposed to another. Thus any protein, structure or even portion of tissue fluid may enter into this relation with any other such entity. The relation is symmetric but not transitive; for example, the plasma membrane of a cell is exposed to both the surrounding extracellular fluid as well as to the cytoplasm but these are not normally exposed one to another (precisely because the plasma membrane is between them). A requirement for the relation of exposure to hold is that the entities exposed are, at a certain level of granularity, in the same location. In other words, these entities need to be near enough; the nearness involved in exposure is vaguer than the standard spatial relation of adjacency [15].

### Sites

Central to our purpose is the localisation of both objects and processes (or of types thereof). The location of processes is important because in order for a process to occur in a given site, its participants must also be found in that site—as has been noted in the particular case of protein interactions. We use *fnd‐in* as a relation between a protein (type) and a site that contains proteins of that type, thus:

$$\begin{array}{lll} & (P-1)(\to (\exists \;x(\wedge (\text{occurs}-\text{in}\;x\;y)(\text{participates}-\text{in}\;z\;x)))\; & \;\\ & (\text{fnd}-\text{in}\;z\;y))\; & \; \end{array}$$

Read: For all y and z, if there is an x such that x occurs in y and z participates in x, then z is found in y.

Paraphrase: The entities participating in a process are found in the site in which the process occurs.

Assuming a representation of anatomy, the specification of a site needs to be as detailed as possible with respect to the available representation. For example, it needs to be that a given protein is found in the extracellular tissue fluid surrounding cells of the atrial myocardium rather than simply in the heart—provided that the representation of anatomy used allows for such distinctions. We assume that the most specific knowledge regarding location is available, even though, in an application context, preprocessing steps may be needed to obtain such information from available data.

In the present context, we consider three types of compartmental sites for which we assume relevant theories of parts and relations; theories from which we use in this presentation only selected fragments: i) tissue portions containing the protein‐producing cells that anchor the subcellular localisation of proteins; linked to the relevant tissues via *ts‐of*; ii) tissue fluid compartments as a case of extracellular tissue fluid; linked to the relevant tissues via *tf‐of*; iii) cardiovascular tissues that supply and drain tissues and organs. Cardiovascular tissues are a special kind of tissue containing a special extracellular fluid, namely blood plasma. Blood plasma is distinct from the tissue fluid of cardiovascular tissue. As a result of this selection, the routes we consider here are cardiovascular routes and we leave aside other compartments (e.g. lymphatics [16]) which may provide alternative routes in a fuller treatment.

It can be useful to keep two important aspects of localisation separable: i) the organ or tissue level location and ii) the type of subcellular localisation of a protein. When we need to separate these aspects, we use *fnd‐in‐tissue* to link a protein to a tissue compartment in which it is found and *fnd‐in‐loc‐type* (not used here) to link proteins to a selected range of types of subcellular and paracellular localisations, respectively *Ltcyt*, *Ltpm*, *Ltec* when the localisation site falls under the category of *cytoplasm*, *plasma‐membrane*, and *extracellular‐space* (or the corresponding fluid compartment).

The appartus for localisation allows us to record facts about where a given protein is found in a range of sites. This range of sites is also applicable to the location of processes. There are indeed two ways in which a protein may be found in a site, both resulting from the occurrence of a process within or near a site. First, a protein is produced *into* a site—as the outcome of a process that places it in that site. Thus, the protein is found in this site. In such cases, we call the site “the post‐production site” of the protein. Second, a protein can translocate from its post‐production site to another site. The latter happens only for proteins that are free to move.

### Post‐production localisation

A protein is produced as a result of a production process, *Production*, that, being a process, involves participants and occurs within a site. Salient participants are the protein product and the producing cell, both of which are related in this order through *prod‐by*. The most salient site in a production process, however, is not the site in which the process occurs but the post‐production site of the protein. We can apply the foregoing apparatus of localisation to record the post‐production sites for proteins. In the present context, we are particularly concerned with proteins found in the tissue fluid of a tissue or in the plasma membrane of (*pm‐of*) the cells forming the tissue structure of that tissue. Generally, *fnd‐in‐postp* relates a protein to a compartment in which the former is found as a final product. We use *postp*_*t*_ to link a protein to the type of subcellular location under which the post‐production site of this protein falls. For example, a protein produced by a cardiac myocyte into its extracellular space is in fact secreted into the tissue fluid of the myocardium and found in this location post‐production, as we understand the notion of location post‐production in the present theory. A protein produced by an arteriolar smooth muscle cell into its plasma membrane is in fact found attached to the cell’s plasma membrane post‐production.

$$\begin{array}{lll} & (P-2)(\to (\exists (y\;z)(\wedge (\text{prod}-\text{by}\;x\;y)(\text{post}p_{t}\;x\;\text{Ltec})\; & \;\\ & (\text{ts}-\text{of}\;y\;z)\;(\text{tf}-\text{of}\;u\;z)))\; & \;\\ & (\text{fnd}-\text{in}-\text{postp}\;x\;u))\; & \; \end{array}$$

(P‐2) reads: For all x and u, if there is a y and a z such that x is produced by y, the post‐production site for x is of the extra‐cellular type, y is the tissue‐structure portion of z and u is the tissue‐fluid portion of z, then x is found in u post‐production.

Paraphrase: An extra‐cellular product is found in the tissue‐fluid portion corresponding to the structure that produces it.

$$\begin{array}{lll} & (P-3)(\to (\wedge (\text{prod}-\text{by}\;x\;y)(\text{post}p_{t}\;x\;\text{Ltpm})\; & \;\\ & (\text{pm}-\text{of}\;u\;y))(\wedge (\text{fnd}-\text{in}-\text{postp}\;x\;u)(\text{att}-\text{to}\;x\;u)))\; & \; \end{array}$$

(P‐3) reads: For all x, y and u, if x is produced by y, the post‐production site for x is of the plasma‐membrane type and u is the plasma‐membrane of y, then x is found in u post‐production and x is attached to u.

Paraphrase: A plasma‐membrane product is found in the plasma‐membrane of its producer post‐production and is attached to it.

These two cases are highly distinctive of the range of possible locations in which proteins may be found for the purpose of characterising their potential interaction. A protein which is output in a fluid compartment is a free‐moving object and may at least move via diffusion within that compartment. A protein found in a plasma membrane is attached to (*att‐to*) it and thus may only move provided the cell to which it is attached is moving as well. For the sake of simplification, or as a constraint to the scenarios we contemplate, we assume that producing cells belong to tissue structures and may not normally move. Thus, in order for protein interactions of the type contemplated here to take place, proteins, or at least one of them, must be able to move freely. Of course, if cells could move, the phenomenon would be similar at the cellular level but we leave the treatment of this case outside of the present scope.

The foregoing has consequences on whether interactions can occur. Fluid compartments are central to localisation because fluids and structures are exposed one to another. Also, any substantial found in a fluid is *ipso facto* exposed to that fluid. In addition, provided it can move within the fluid—an assumption we will make here—a substantial is potentially exposed to anything exposed to the fluid. Equivalently, it is exposed to anything to which the fluid is exposed. This is the case when a hormone has reached a fluid compartment that bathes a receptor‐presenting tissue structure. Conversely, if a protein is localised in the plasma membrane of a cell, that protein is exposed to anything to which the plasma membrane of that cell is exposed, in particular the tissue fluid surrounding that cell. With similar provisions, given the symmetry of *exp‐to*, if two substantials are found in the same fluid compartment (fluid portions, *Fp*), then they are exposed to each other.

(P−4)(→(∃y(∧(tf−ofxy)(ts−ofzy)))(exp−toxz))

(P‐4) reads: For all y and z, if there is a y such that x is the tissue‐fluid portion of y and z is the tissue‐structure portion of y, then x is exposed to z.

Paraphrase: The tissue‐fluid and tissue‐structure portions of a tissue are exposed one to the other.

(P−5)(→(∧(fnd−inxy)(Fpy))(exp−toxy))

(P‐5) reads: For all x and y, if x is found in y and y is a fluid portion, then x is exposed to y.

Paraphrase: An entity is exposed to the fluid portion in which it is found.

$$\begin{array}{lll} & (P-6)(\to (\exists \;y(\wedge (\text{fnd}-\text{in}\;x\;y)(\text{Fp}\;y)(\text{exp}-\text{to}\;y\;z)))\; & \;\\ & (\text{exp}-\text{to}\;x\;z))\; & \; \end{array}$$

(P‐6) reads: For all x and z, if there is a y such that x is found in y, y is a fluid portion and y is exposed to z, then x is exposed to z.

Paraphrase: An entity is exposed to whichever entity is exposed to the fluid portion in which it is found.

$$\begin{array}{lll} & (P-7)(\to (\exists \;y(\wedge (\text{prod}-\text{by}\;x\;y)(\text{post}p_{t}\;x\;\text{Ltpm})\; & \;\\ & (\text{exp}-\text{to}\;y\;z)))(\text{exp}-\text{to}\;x\;z))\; & \; \end{array}$$

(P‐7) reads: For all x and z, if there is a x such that x is produced by y, the post‐production site for x is of the plasma‐membrane type and y is exposed to z, then x is exposed to z.

Paraphrase: A plasma‐membrane product is exposed to whichever entity is exposed to its producer.

$$\begin{array}{lll} & (P-8)(\to (\exists \;y(\wedge (\text{Fp}\;y)(\text{fnd}-\text{in}\;x\;y)(\text{fnd}-\text{in}\;z\;y)))\; & \;\\ & (\text{exp}-\text{to}\;x\;z))\; & \; \end{array}$$

(P‐8) reads: For all x and z, if there is a y such that y is a fluid portion, x is found in y and z is found in y, then x is exposed to z.

Paraphrase: Two entites in the same fluid portion are exposed one to the other.

Free‐to‐move biological structures may be found in many locations in the body. This is true for proteins that may translocate from their site of production. When a protein is free to move, it can be found in any compartment it can access (*acc*) from the site in which it is initially found. It may be any site or compartment provided a suitable compartmental connection exist. Such a connection can be mediated, e.g. passing through fluid‐containing structures, so long as it allows for an adequate kind of translocation for the protein. In general, this is the case when compartments communicate according to a translocation modality of which a translocating object is capable.

### From protein interactions to routes

We use *Interaction* in order to declare an interaction between (*int‐btw*) two kinds of protein. We use the general *participates‐in* in order to relate a kind of protein involved in an interaction to that interaction. A requirement for a protein interaction to occur is that the interacting proteins be exposed to each other. Based on subcellular location, it is possible to determine a first classification of interactions. The requirements for colocation are trivially met when proteins have the same post‐production site and are exposed to each other. A layer of complexity is added when one of the proteins is attached to the plasma membrane of the producing cell as, in these cases, membrane crossing may be added to cytoplasmic diffusion. A special case, when both proteins are attached and protrude externally from the plasma membrane, may lead to a requirement of cell translocation in order to allow protein interaction. Cases in which one protein is cytoplasmic may involve complex molecular pathways. We leave these cases aside. While these are, of course, of interest, not least because they are key to autocrine and juxtacrine interactions, there is no room here for a full treatment. We focus instead on those cases involving only direct interactions requiring the translocation of proteins, translocations that are moreover between anatomical locations that are potentially far apart.

The endocrine scenario corresponds to an interaction in which one protein’s subcellular localisation is in the extracellular space and the other attached to a plasma membrane and the post‐production sites of the two proteins are far apart. In those cases, one protein, the ligand (which ligand may further play the role of a hormone), is free to move while the other, the membrane‐bound receptor, is not. Such cases, therefore, require a process of translocation in order for the interaction to occur. We say that a translocation subtends an interaction when that is the case (*trans‐subtends‐int*):

$$\begin{array}{lll} & (P-9)(\to (\exists (x\;y)(\wedge (\text{int}-\text{btw}\;x\;y\;z)(\text{post}p_{t}\;x\;\text{Ltec})\; & \;\\ & (\text{post}p_{t}\;y\;\text{Ltpm})))(\exists \;t(\text{trans}-\text{subtends}-\text{int}\;t\;z)))\; & \; \end{array}$$

(P‐9) reads: For all z, if there is a x and a y such that z is an interaction between x and y, the post‐production site for x is of the extra‐cellular type and the post‐production site for y is of the plasma‐membrane type, then there is a t such that t is a translocation that subtends z.

Paraphrase: A translocation is required for an interaction between an extra‐cellular product and a plasma‐membrane interact.

**Figure 1:**
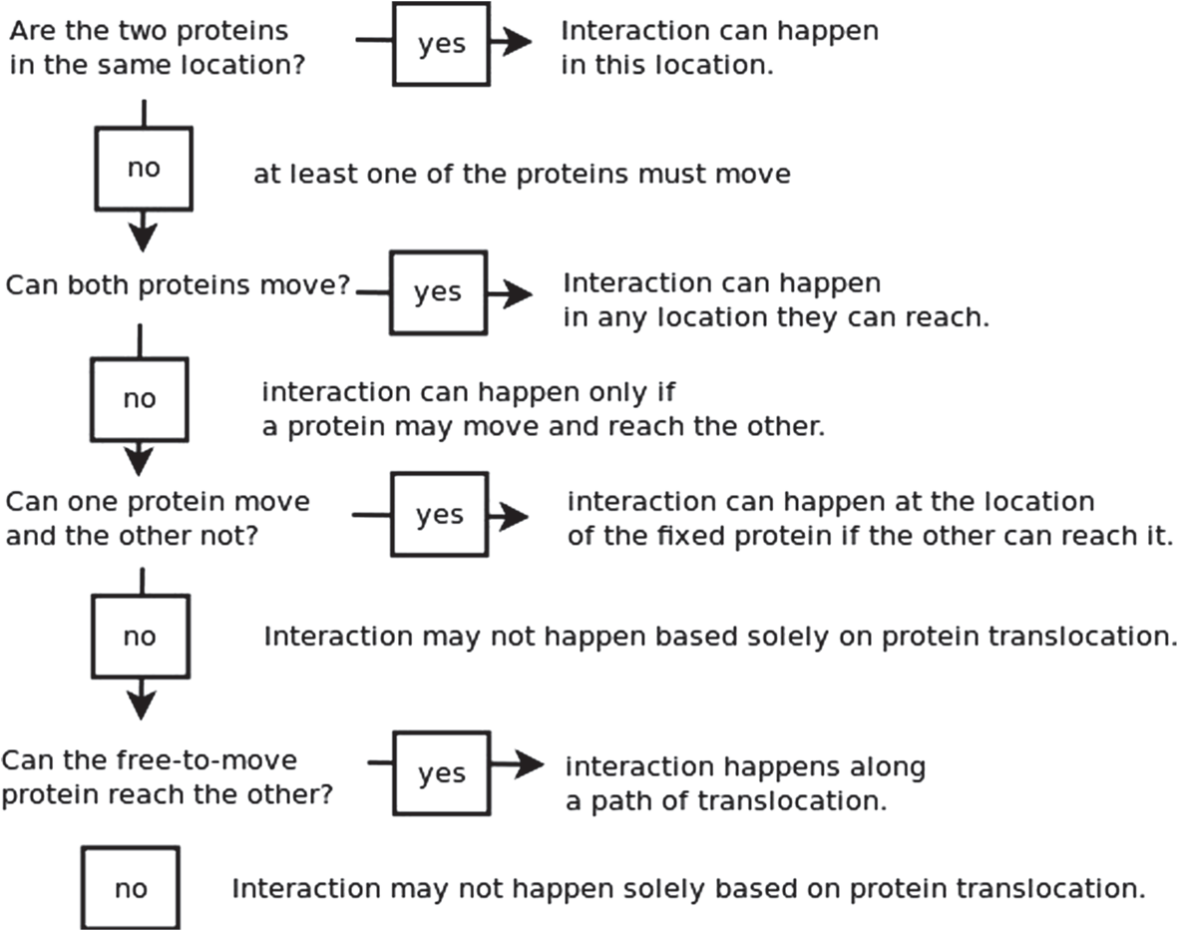
**Decision procedure.** Decision procedure for the selection of interactions as being subtended by a translocation route.

At this point, given a pair of proteins (protein types), it is possible to select possible scenarios on the basis of whether the interaction requires a subtending route by using, as part of the heuristic, the subcellular localisation types of post‐production sites of proteins. We assume that only extracellular products (proteins secreted by cells) may move and not those circumscribed to the cytoplasm nor those normally attached to a plasma membrane. There are then two possible cases: 1) both may translocate and 2) only one may translocate. In the first case, an interaction can occur in any location that may be reached by both proteins. In the second case, one of the proteins must reach the site of the other in order to interact with it—endocrine interactions are of this sort. Figure 1 illustrates the overall descision procedure. It is important to consider that these heuristic elements, that are tied to subcellular localisations of proteins, are not sufficient conditions for interactions to actually take place. In practice, potential translocation routes must actually be patent. However, these elements indicate necessary conditions, and therefore, we may use the subcellular localisation of proteins to screen potential interactions and the types of transport phenomena that may subtend them.

While we assume permissible fluid barriers throughout the body, given that a potential communication route is endowed with a modality according to which it can be followed, it is important to reflect the expectation that a translocating object may use such a route under one modality or another. In effect, to a forthcoming treatment of accessibility in terms of affordances provided by structures, we can substitute a treatment in terms of a range of capabilities possessed by the biological agents susecptible of translocations—here, proteins.

### Communication

Translocating objects can access compartments when these compartments communicate according to a given translocation modality. A necessary condition for two portions of the same or distinct compartments to be directly communicating is that they be contiguous, or connected (*CONN*). Hence, two compartments communicate when i) they are connected and ii) allow for certain agents found in them to interact. The ternary relation *COMM*^3^ holds when two compartments communicate following a specified modality in the form of a process type (in the third argument place of the relation). *COMM*^3^ is commutative in its first and second positions (filled by compartments) and defines a transitive binary relation when its third argument is fixed.

The modalities of communication we consider in the present context are kinds of translocation in a fluid. Translocations can be characterised as being translocation of an object (*tr‐obj*, an inverse specialisation of *participates‐in*), from an initial location (*from‐loc*) to a final location (*to‐loc*) along a given route (*tr‐site*, not used here). Consistently with BFO [13], a portion of fluid medium occupying a site is part of that site and a translocation process happens in that site or in that medium, interchangeably, according to various modalities. Here we consider a small number of modalities corresponding to process‐types specialising *Translocation*: i) *advection*, *Adv*: a translocation process whereby an entity is moved in a fluid medium as a result of the movement of the fluid medium itself; ii) *diffusion*, *Dif*: a translocation process whereby an entity moves in a fluid medium without that movement resulting from the movement of the medium itself, iii) *convection*, *Cnv*: a translocation combining diffusion in advective flow.

**Figure 2:**
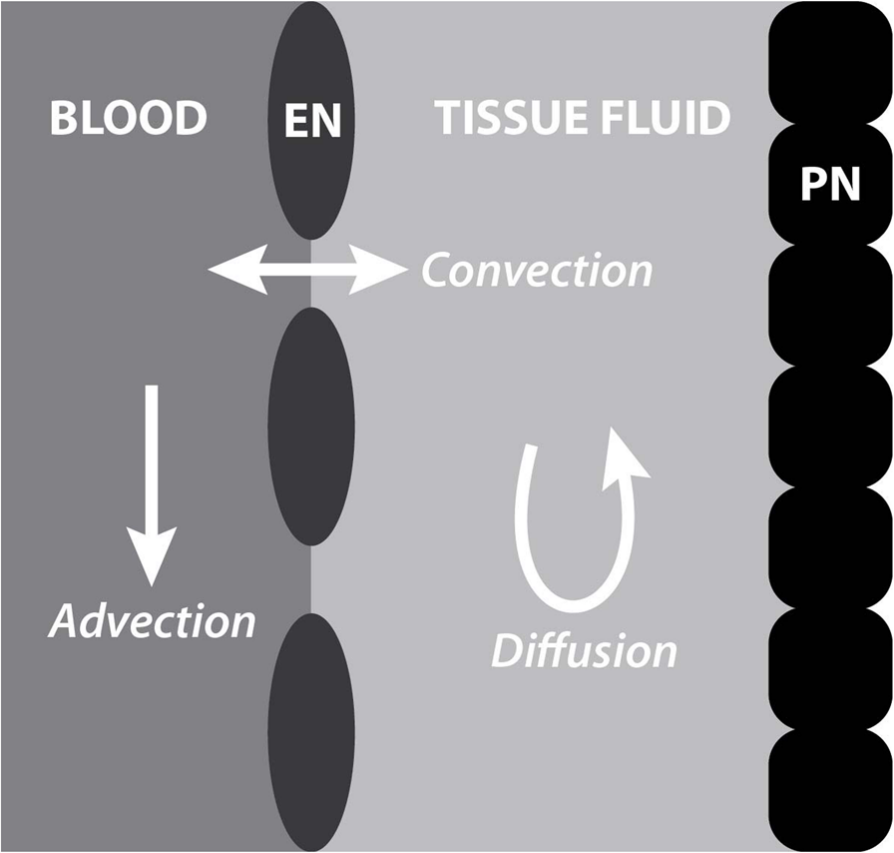
**Fluid compartmental communication.** Basic routes and modes of communication within and across fluid compartments: advection in blood, diffusion in tissue fluid, and convection between blood and tissue fluids. EN:endothelium, PN: parenchyma.

It is part of our domain knowledge axiomatisation that compartments of certain types communicate according to certain modalities. In particular, 1) connected portions of tissue fluid (TF) diffuse into each other, 2) connected portions of the cardiovascular system (CVS) advect in the direction of blood flow, and 3) connected portions of TF and CVS are such that they allow for convection one into the other (this is depicted in Figure 2).

Because paths between TF portions may go through circulation, it is important to finely articulate TF‐to‐CVS communication. Indeed, TF diffuses into CVS. Not any portion of TF, however, diffuses into any portion of CVS. Those that do so need to be connected. Such connections exist in vasculatures of organs at the level of capillaries. Thus, the TF of an organ connects to, and supports convection to and from, the capillaries of this organ (*cap‐of*):

$$\begin{array}{lll} & (P-10)(\to (\exists \;z(\wedge (\text{tf}-\text{of}\;x\;z)(\text{cap}-\text{of}\;y\;z)))\; & \;\\ & (\text{COM}M^{3}\;x\;y\;\text{Conv}))\; & \; \end{array}$$

(P‐10) reads: For all x and y, if there is a z such that x is the tissue‐fluid portion of z and y is the (aggregated) capillaries of z, then x and y communicate convectively.

Paraphrase: The tissue‐fluid portion of an organ (more generally, of a portion of tissue) and its capillaries communicate advectively.

While in the CVS, advective communication follows the direction of blood flow, since the cardiovascular system is a closed circuit, we retain the more general fact according to which any two CVS portions are accessible one from the other via some route. This fact corresponds to the assumptions made in modelling transport phenomena according to which these routes exist and blood plasma is well mixed.

$$\begin{array}{ll} \left(P-11)(\to (\wedge (\text{cvs}\;x)(\text{cvs}\;y))({\text{COMM}}^{3}\;x\;y\;\text{Adv})\right) & \; \end{array}$$

(P‐11) reads: For all x and y, if x is a portion of CVS and y is a portion of CVS, then x and y communicate advectively.

Paraphrase: Portions of the cardiovascular system communicate advectively.

Cardiovascular vessels are tissues and thus, as a particular case, their lumenal blood plasma compartments communicate via convection with their TF compartments:

$$\left(P-12)(\to (\wedge (\text{cvs}\;x)(\text{tf}-\text{of}\;y\;x))\;(\text{COM}M^{3}\;x\;y\;\text{Cnv})\right)$$

(P‐12) reads: For all x and y, if x is a portion of CVS and y is the tissue‐fluid of x, then x and y communicate convectively.

Paraphrase: Portions of the cardiovascular system communicate convectively with their tissue fluid.

The above applies to virtually all vessel‐like portions of the CVS except for capillaries which communicate with the body fluid compartment of the organs they supply—a fact registered above.

### Putting it togethers

If a protein is found in a compartment and that compartment communicates with another compartment according to a modality of translocation that the protein is capable of, then the protein can access the latter compartment. We use *CMTP*_*t*_ (read ‘can move through path type’) to relate a (type of) protein to a type of translocation of which it is capable. Such a relation supports, in effect, a classification of agents (as advective, diffusive, but also secreted and so on) and allows identifying and selecting relevant candidate routes or portions thereof.

$$\begin{array}{lll} & (P-13)(\to (\exists (y\;w)(\wedge (\text{fnd}-\text{in}\;x\;y)(\text{COM}M^{3}\;y\;z\;w)\; & \;\\ & (\text{CMT}P_{t}\;x\;w)))(\text{acc}\;x\;z))\; & \; \end{array}$$

(P‐13) reads: For all x and z, if there is a y and a w such that x is found in y, y communicates with z according to w and x can move through a path of type w, then x can access z.

Paraphrase: An entity can access any site that is communicating, according to a given modality of communication, with a site in which that entity is found provided such entity can move through translocation paths affording the relevant mode of communication.

As a recursive step, if a protein can access a compartment and that compartment communicates with another compartment according to a modality of translocation that the protein is capable of, then the protein can access the latter compartment.

$$\begin{array}{lll} & (P-14)(\to (\exists (y\;w)(\wedge (\text{acc}\;x\;y)(\text{COM}M^{3}\;y\;z\;w)\; & \;\\ & (\text{CMT}P_{t}\;x\;w)))(\text{acc}\;x\;z))\; & \; \end{array}$$

(P‐14) reads: For all x and z, if there is a y and a w such that x can access y, y communicates with z according to w and x can move through a path of type w, then x can access z.

Paraphrase: An entity can access any site that is communicating, according to a given modality of communication, with another site that this entity can access provided such entity can move through paths affording the relevant mode of communication.

Let us assume now that anything which may access a compartment may be found in that compartment:

(P−15)(→(accxy)(fnd−inxy))

(P‐15) reads: For all x and y, if x can access y, then x is found in y.

Paraphrase: Any site that is accessible to an entity is a site in which this entity is (can be) found.

It suffices, then, that a substantial can access a compartment to which is exposed another substantial for the two to be exposed one to another:

(P−16)(→(∧(accxy)(exp−tozy))(exp−toxz))

(P‐16) reads: For all x, y and z, if x can access y and z is exposed to y, then x is exposed to z.

Paraphrase: An entity is exposed to anything to which what it can access is exposed.

Since both substantials are exposed to one another, they can interact with one another. Hence, finally, it follows that if there is a route which is accessible to a protein from its post‐production site to a site where it can become exposed to a potential interactor, then their interaction may occur. This possibility, however, is depedent upon the existence of a route. The relation *CIVSR*, read ‘can interact with via some route’, holds of two proteins when the first can access a compartment to which is exposed the second.

(P−17)(→(∧(accxz)(exp−toyz))(CIVSRxy))

(P‐17) reads: For all x, y and z, if x can access z and y is exposed to z, then x can interact with y via some route.

Paraphrase: An entity can interact with another when one can access a site to which the other is exposed.

### Route construction

If we assume only a knowledge base of tissue structures, tissue fluids, and of cardiovasculature, the above theory allows the inference of the existence of routes of communication for protein interactions provided that a minimal amount of specific knowledge regarding the pair of interacting proteins is available. This minimal knowledge consists of grounds facts pertaining to i) post‐production locations of proteins (or a combination of their tissue structure and subcellular type location), ii) the translocational capabilities of these proteins (whether they can advect, diffuse or convect)—independently of whether they are or not free‐moving objects, as this may be inferred. In the endocrine scenario, the route of communication for an interaction between proteins can be characterised by analogy to an hypothetical translocation subtending that interaction—a translocation whose existence is guaranteed by (P‐9). Such a translocation is that of the hormone from its post‐production site to the tissue fluid compartment to which is exposed the plasma membrane of the cells to which receptors are attached:

$$\begin{array}{lll} \; & (P-18)(\to (\exists (y\;z\;v\;m\;o)(\wedge (\text{int}-\text{btw}\;x\;y\;z)\; & \;\\ & (\text{trans}-\text{subtends}-\text{int}\;t\;z)(\text{fnd}-\text{in}-\text{postp}\;x\;u)\; & \;\\ & (\text{post}p_{t}\;x\;\text{Ltec})(\text{fnd}-\text{in}-\text{postp}\;y\;v)(\text{pm}-\text{of}\;v\;m)\; & \;\\ & (\text{tf}-\text{of}\;n\;o)(\text{exp}-\text{to}\;v\;n)))(\wedge (\text{tr}-\text{obj}\;t\;x)\; & \;\\ & (\text{from}-\text{loc}\;t\;u)(\text{to}-\text{loc}\;t\;n))).\; & \; \end{array}$$

(P‐18) reads: For all x, t, u and n, if there is a y, a z, a m, a v and a o such that z is an interaction between x and y, t is a translocation subtending z, x is found in u post‐production, the site of post‐production of x is of the extra‐cellular type, y is found in v post‐production, v is the plasma‐membrane of m, n is the tissue‐fluid of o, and v is exposed to n, then x is the translocated object in t, t is from u and t is to n.

Paraphrase: When a protein produced into an extra‐cellular compartment interacts with one produced into a plasma‐membrane, a translocation subtending their interaction is a translocation of the first protein from its post‐production site to the tissue‐fluid to which is exposed the plasma‐membrane into which is produced the second protein.

### Route elicitation algorithm (endocrine case)

We now give an overall algorithm for the elicitation of routes of communication of protein interaction under the foregoing assumptions. Details are illustrated in the endocrine case. In steps 1.2.1 and 2.3.3, relevant heuristic rules are selected from a set that encodes domain specific knowledge; this allows applying the algorithm to various scenarios. Occurrences of such selection are indicated by the use of the phrase ’one of’ enclosed within parentheses. In this application of the algorithm, step 1.2.1 leads to specifying an endocrine case; step 2.3.3 further specifies the scenario according to the types of locations bounding the hypothetical route considered (here, a route joining two distant tissue fluid portions). 

1. Make hypothetical interaction 

1.1 Step: Define a term for interaction between A and B, I{A,B}

1.1 Step: Specify Interaction, 

1.2 Heuristics (one of):

if A can move and B cannot, then A must move (endocrine case)

1.2 Endocrine case: Identify roles 

1.2.1 Hormone is the translocation capable protein

1.2.1 Receptor is the fixed target

2. Make hypothetical route (endocrine case) 

2.1 Step Define route: as the route the hormone must/may follow in order to reach its receptor, R{A,B}

2.1 Step Forward rules are fired: 

2.2 assert that the route subtends the interaction

2.2 assert that the route is from the post‐production location of the hormone

2.2 assert that the route is to the post‐production location of the receptor

2.2 OPT: modality (e.g. CVS, lymphatics...)

2.1 Step Resolve route: 

2.2 Absolute start (typically, tissue fluid where the hormone is secreted)

2.2 Absolute end (typically, tissue fluid to which the receptor is exposed)

2.2 Heuristics (one of): If starts is in tissue fluid and ends in tissue fluid, the hormone needs to reach blood and advects through CVS

2.2 find CVS entry point (closest accessible CVS portion to absolute start)

2.2 CVS exit point (closest accessible portion to absolute end)

2.2 CVS route (delegated)

3. Construct route: 

3.1 Construct route from absolute start to CVS entry (leg1)

3.1 Construct route from CVS entry point to CVS exit point (leg2)

3.1 Construct route from CVS entry exit point to absolute end (leg3)

3.1 Assert the extension of R{A,B} as the concatenation of (leg1 leg2 leg3)

The steps of construction are left open. A route can be associated to a series of compartments that are traversed by a substantial following this route—from the absolute start to the absolute end of the route. Backward inferencing allows to find elements in this series. It is not immediate, however, to produce the complete series itself. Rather, the actual construction of the routes is partly procedural and dependent upon the implementation of a system. We turn to this matter now.

## Implementation and use case

### Implementation

To support our knowledge representation and reasoning tasks, the prototype implementation of the theory presented in this paper is developed using the PowerLoom system [17]. PowerLoom, PLM hereafter, allows for knowledge representation using the logic‐based KIF language [18] which provides suitable expressivity for our theory. We can thus assert ground facts for our theory and query the resulting knowledge base using classification and rule‐based inferencing. This theory has access to a knowledge base containing: i) a representation of the cardiovascular system connectivity in terms of segments and their connections, ii) a representation of a number of organs and regions together with their vascular arterial supply and drainage. These are built by harvesting the Foundational Model of Anatomy [19], in the case of ii) above, and following a process of automated expansion and curation, in the case of i) above. Additionally, the knowledge base is expanded when loaded to include tissue fluid compartments of the known vascularised regions together with inferred connections between these tissue fluid compartments and the relevant capillaries.

The resulting knowledge base contains hundreds of thousands of ground facts. Its representation of the human cardiovascular system and associated tissue fluid compartments creates a substantial multigraph of over ten thousand nodes. In this contxt, while inference capabilities of PLM remain satisfactory for many tasks, the navigation of the resulting connectivity graphs is not always efficient. For this reason, we implement graph traversal using the JUNG2 java library. The integration is done using Clojure [20], a dialect of Lisp, from which both PLM (also released as a java library) and JUNG2 can be combined. Thus, our algorithm for the construction of routes is implemented in Clojure; it relies on the inferencing capabilities of PLM in order to construct graph structures that may then be processed using JUNG2 methods.

A path may be defined logically at the level of PLM and the graph library allows us to construct the corresponding extent for this path. In effect, this construction associates a list of compartments traversed when following a logically defined route. In the simplest cases, this series corresponds to a simple Dijkstra shortest path computation. In more complex cases, including when (initial and final) locations are scattered, the thought out route may in fact be an entire portion of the graph—consider, for example, the tissue fluid compartments of both lungs as a location in its own right in contrast to that of one lung only.

### ANP‐ANPr interaction case study

In the remainder of of this section, we illustrate the use of in the context of the described prototype implementation in the context of a specific case study. This case study consists in eliciting a candidate vascular route of communication between the site of production of a hormone, namely ANP, and a site in which a receptor for ANP, namely ANPr, is located. ANP is normally produced and stored in the cardiac atrial myocytes. It is secreted into tissue fluid in reaction to the stretching of the atria due to increased volume. ANP has a vasolidator action and is thought to be involved in diuretic regulation. Organ locations of receptors, ANPr, include the kidneys, renal arterioles, lungs and bone marrow. ANPr is attached to the plasma membrane of its producing cells, and the majority of its polypeptide chain is located in the surrounding tissue fluid. In order to illustrate the application of the theory and prototypical implementation presented, we demonstrate the kinds of results we obtain from our system. We ignore the process of exocytosis that releases ANP from cardiac myocytes and focus on release to the bloodstream and accession to compartments exposed to receptors. We do not consider the possibility of routes involving the special case of direct translocation from the tissue fluid compartment of an atrium to its chamber [21].

We apply our algorithm to the construction of one possible route of communication between two specific locations as our implementation takes advantage of the Dijkstra algorithm searching for the shortest path between two discrete nodes in a graph. We focus on this base case; providing more or alternate routes consists in performing similar operations as in the base case and combining their results. We compute an intuitively simple route of communication for an interaction between ANP produced in the tissue fluid of the right atrium and ANPr in the smooth muscle of the left renal arterioles.

The knowledge base used in this scenario includes: 1) a representation of cardiovascular tissue connectivity, 2) definitions for TF compartments of all represented tissues and 3) definitions for certain tissue structures, such as endothelium for all CVS portions and smooth muscle for all portions except capillaries and cardiac chambers. Two graphs are constructed from the results of queries to the knowledge base: 1) a graph of the CVS whose edges correspond to CVS portions and nodes to boundaries between them; 2) a graph connecting tissue fluid compartments to their corresponding CVS connections whose nodes are fluid compartments and capillaries and whose edges are boundaries between these. Thus, in particular, there is no connection between the tissue fluid of the atria and their chamber, ruling out a corresponding type of route mentioned above.

The knowledge base contains no pre‐existing knowledge of proteins. Thus, a minimal number of facts needs to be asserted in order to define the proteins in this scenario and to record their relationship to the most specific locations at the level of granularity corresponding to locations predefined in the knowledge base. Barrier phenomena are not represented and we assume that ANP has the necessary physicochemical properties to pass through capillary walls without further elaboration—a realistic assumption in this case. Thus, also, we assert that ANP is capable of following diffusive, advective and convective routes. 

 (*Protein* anp) ; ANP is a type of protein

 (*fnd‐in‐postp* anp TF‐7096) ; ANP is found, post‐production, in the right atrium’s tissue fluid

 (*Protein* anpr) ; ANPr is a type of protein

 (*fnd‐in‐postp* anpr SM‐ARTL‐CVS‐25) ; ANPr is found, post‐production, in the smooth muscles of arterioles of the left kidney

 (*CMTP*_*t*_ anp adv) ; ANP can advect

 (*CMTP*_*t*_ anp dif) ; ANP can diffuse

 (*CMTP*_*t*_ anp cnv) ; ANP can convect

The addition of the above facts is sufficient for the system to infer triage along the lines illustrated in Figure 1. On the basis of the inferred subcellular location, it can be automatically established that ANP, but not ANPr, may translocate. In a subsequent step, path constructors are initiated to generate a path as a list of compartments in serial connection between the post‐production sites of the proteins.

The process of route elicitation is initiated by calling a function which takes as arguments the two proteins. This function generates an hypothetical interaction and a subtending translocation, when its existence is provable. The process continues along the lines delineated by the route elicitation algorithm. The raw output of such a process consists of a list of symbols for compartmental sites presented in an order that follows the shortest route of translocation allowing ANP produced in the right atrium to reach ANPr in the left renal arterioles, as shown below: 

("TF‐7096" "BND‐12078" "CAP‐CVS‐448" "VNL‐CVS‐448" "CVS‐2076" "CVS‐2075" "CVS‐2074" "CVS‐2072" "CVS‐1333" "RV" "PC" "LH" "CVS‐2407" "CVS‐2573" "CVS‐2657" "CVS‐3780" "CVS‐4288" "CVS‐4707" "CVS‐4709" "CVS‐4711" "CVS‐4713" "CVS‐4715" "CVS‐4716" "CVS‐4650" "CVS‐4719" "CVS‐4770" "CVS‐4819" "CVS‐4868" "CVS‐4917" "CVS‐4964" "CVS‐5011" "CVS‐5058" "CVS‐5105" "CVS‐5152" "CVS‐5199" "CVS‐5246" "CVS‐5293" "CVS‐5295" "CVS‐5297" "CVS‐5344" "CVS‐5391" "CVS‐5438" "CVS‐5485" "CVS‐5532" "CVS‐5579" "CVS‐5618" "CVS‐5657" "CVS‐5659" "CVS‐5661" "CVS‐5673" "CVS‐5685" "CVS‐5812" "CVS‐5864" "CVS‐5866" "CVS‐5868" "CVS‐5870" "CVS‐5871" "ARTL‐CVS‐25" "BND‐12524" "TF‐ARTL‐CVS‐25")

**Figure 3:**
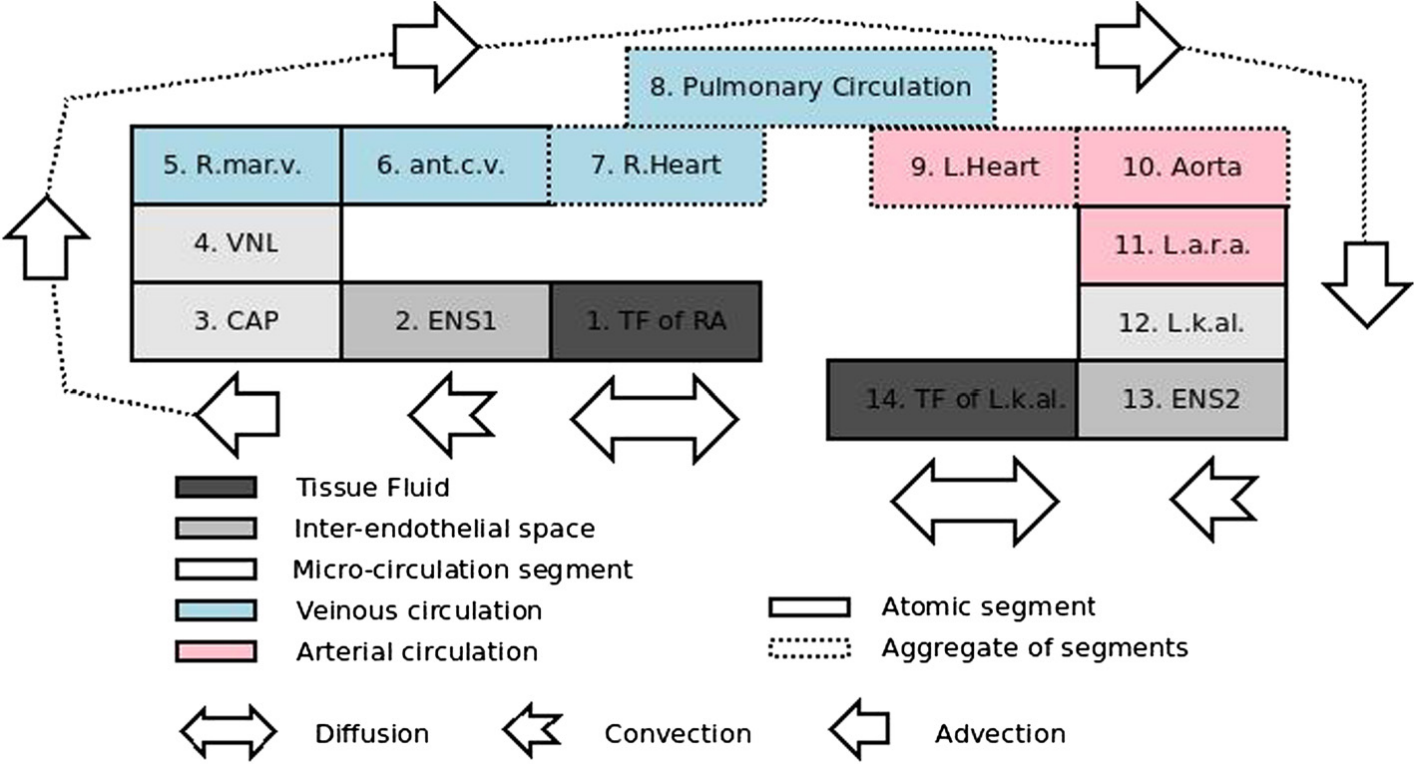
**Condensed elicited path for the ANP‐ANPr interaction case study.** The path for the translocation of ANP from the right atrium to the left renal arterioles is represented in its condensed form. Segments belonging to the same vessel (e.g. segments of the aorta) are aggregated in a single box. The boxes labelled with main vessels (blue and pink) represent mere portions of these vessels (R. marg. v. = right marginal vein, ant. c. v. = anterior circulatory vein, R.Heart = right heart, L.Heart = left heart, L.a.r.a. = Left accessory renal artery). The microcirculatory elements are: VNL for venules of the right atrium, CAP for capillaires of the right atrium, and L.k.al for left kidney arterioles. ENS stands for inter‐endothelial space and the rest follows conventions already explained. The illustration also shows the types of physiological communication involved.

These terms represent portions of fluid compartments or of the cardiovascular system that are followed when following the route. Some of these are atomic segments, while others represent defined subgraphs whose extension can be unfolded programmatically. It would be impractical to follow the above path compartment by compartment, but we can summarise the path by taking compartments that are portions of the same CVS vessel to be equivalent (Figure 3). The following has been edited manually to such an effect on the basis of information about the relevant list members found by querying the knowledge base: 

C1: Tissue fluid of the right atrium (RA), TF‐7096

C2: Inter‐endothelial space between C1 and the capillaries of RA, BND‐12078

C3: Capillaires of RA, CAP‐CVS‐448

C4: Venules of RA (at junction to right marginal vein), VNL‐CVS‐448

C5: Segment of right marginal vein, CVS‐2076

C6: Segment of anterior cardiac vein, CVS‐2075

C7: Right heart—strictly speaking only including a 3‐segments portion of the RA chamber (CVS‐2074, CVS‐2072, CVS‐1333) and the right ventricle (RV)

C8: Pulmonary circulation, PC, which can be unfolded as a subgraph of the cardiovacular system

C9: Left heart, LH

C10: Portion of the aorta (44 segments from CVS‐2407 to CVS‐5870) from the left ventricle to the left accessory renal artery

C11: Portion of the left accessory renal artery (CVS‐5871)

C12: Arterioles of the left kidney, ARTL‐CVS‐25

C13: Inter‐endothelial space between C12 and the TF of the left kidney, BND‐12524

C14: TF of the left kidney, TF‐ARTL‐CVS‐25

If we abstract further, we obtain the overall physiological communication footprint for this route of communication by taking compartment transporting ANP according to the same modality as equivalent. The result may be rendered as the following list: 

1. Diffusion: C1

2. Convection: C2

3. Advection: C3‐12

4. Convection: C13

5. Diffusion: C14

Hence, the above computations result in the series of types of physiological processes subtending the interaction of ANP produced in the right atrium with ANPr receptors found in the left renal arterioles, given an elicited translocation route.

## Results and discussion

In this work, we presented a theory of physiological transportation along anatomical routes for protein interactions and a prototypical implementation using this theory. This implementation provides the symbolic computation of a translocation route, in the scenario used here: from the site of secretion of a hormone to the site of interaction with its receptor. Such computation, which uses the presented theory, relies on the declaration of location data about the sites where ligand and receptor are produced, and a knowledge base of connectivity between relevant anatomical structures and compartments.

We currently do not represent physicochemical attributes associated with biological structures that play a major role in determining the likelihood of particular translocations to occur (e.g that a specific protein gain access to the TF in some specifc tissue portion). Furthermore, certain physiological effects are ignored. For example, we regard TF as only allowing for diffusion, ignoring that external mechanical stresses imposed on the TF could also allow advective forms of translocation. Moreover, the knowledge base could be extended to include connectivity in more body systems (digestive, urinary, respiratory and so on); thus our ANP use case could be extended to interaction in renal tissues. Also, the theory takes only into consideration kinds of translocation as modalities of communication and the work could be extended to include mechanical (e.g. stress), electrophysiological and other modes of communication; thus, our ANP scenario could be extended to take into account mechanical trigger mechansims for ANP secretion. Finally, the present paper leaves aside more local, in particular, intracellular and transmembrane mechanisms and these could be included in extended physiological pathways so as to obtain full length, detailed communication pathways. Refinements and developments in these direction are topic for future work.

While we have not approached the following aspect in the present paper, it is worth noting that our knowledge representation is readily mapped to existing and well established ontologies that are widely used in the annotation of biomedical data, models and related resources. In particular, part of our knowledge base for gross anatomy has been mapped to the Foundational Model of Anatomy *ab initio*, subcellular and paracellular locations used in the present paper are found in the GO Cell Component ontology [22] and cells in the CellType ontology [23]. This approach opens two avenues of application for our work.

The first avenue consists in taking further concrete steps towards a knowledge‐rich capitalisation on the wealth of data scattered in multiple specialised databases and services that use the mentioned and related ontologies for the purpose of annotations. For example, ArrayExpress [24] for anatomy‐mapped gene expression, HumLoc [25] for subcellular protein localisation mapped to GO Cell Component terms, and IntAct [26] for protein‐protein interaction data (PPI). By acquiring gene expression localisation data and interaction constraints from bioinformatics databases or services, it becomes feasible to automatedly elicit and propose concrete anatomical routes involved in cellular communication processes. The integration of such resources would also allow to start the process of route elicitation from region‐specific diseases, and find genes expressed in those regions whose product is interacting with other gene products in other locations. Ontological definitions of routes could be deposited in a database with standardised accessors. However, as the computation of routes is dependent on the connectivity knowledge base available, concrete paths may be less persistent. In such cases, the prototypical implementation described here could lead to the deployment of a service‐based application providing updated route specifications. As an indication of possibilities, the cardiovascular part of our knowledge base and representation mapped to parts of SNOMED and ICD‐10 has already been put to use in the calculation of an anatomical distance metrics for diseases of the cardiovascular system [27].

The second avenue of application for our work comes in support of ongoing efforts of communities in computational physiology, e.g., RICORDO [28], pharmacometrics, e.g., DDMoRe [29], and drug discovery, e.g., OpenPHACTS [30], to integrate semantic metadata about data and modelling resources (CellML, SBML and pharmacological models as well as clinical data). This is done through the application of a shared standard of multiscale anatomy knowledge to the knowledge management of these resources. In this context, the work could be applied to the identification of physiological relations between data and models annotated with ontologies. Specifically, given ontological annotations linking data and models to communicating sites, it would become possible to identify resources that are physiologically‐related via their annotations to sites along the anatomical connectivity route that conveys a given communication process. With this perspective in mind, our larger work includes the development of a front‐end to systems such as the one presented in this paper for the visualisation and curation of annotated data within their anatomical and physiological context, [31].

**Figure 4:**
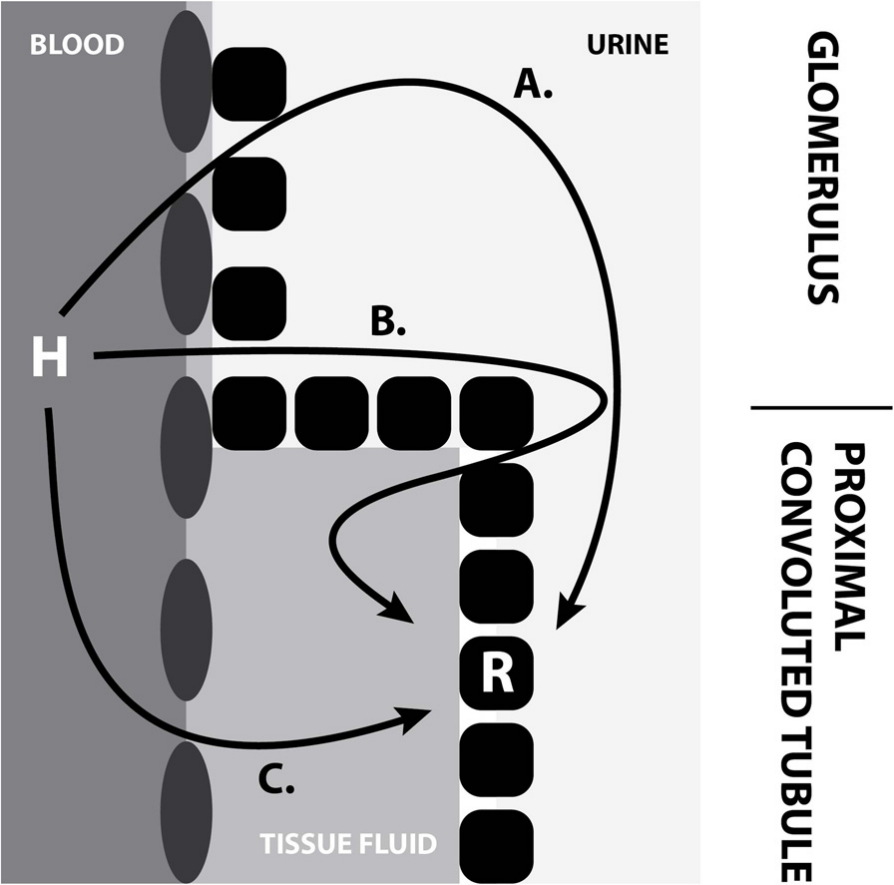
**Nephron scenario.** An extension on the ANP scenario involving the nephron in the urinary tract and the elicitation of candidate locations for ANPr on the basis of accessible locations for ANP. The arrows indicate possible routes (by extension, types of routes) that a hormone (H) may follow in order to reach a receptor (R). Oblong shapes represent endothelial cells of renal capillaries and rounded square shapes represent epithelial cells in the nephron.

The early results presented here provide an indication and motivation for an extended curation of a compartmental connectivity knowledge representation. A more detailed knowledge base of compartment connectivity would improve the breadth of the types of data and models that could be related physiologically, as well as potentially support the automated generation of new hypotheses for experimental investigation. An example for the latter scenario is illustrated in Figure 4. In this scenario, a more detailed connectivity KB permits the querying of potential routes of communication linking blood in the renal capillaries to epithelial cells in the nephron. Epithelial cells lining the proximal convoluted tubule are known to express ANPr [32]. Given that information about ANPr expression, as well as an extended knowledge base of renal compartment connectivity, our method is able to determine that there are indeed three distinct routes by which ANP can reach its receptor (routes A‐C in Figure 4). These hypothetical alternative communication paths are scientifically sound and not experimentally confirmed as yet (see Acknowledgements).

**Figure 5:**
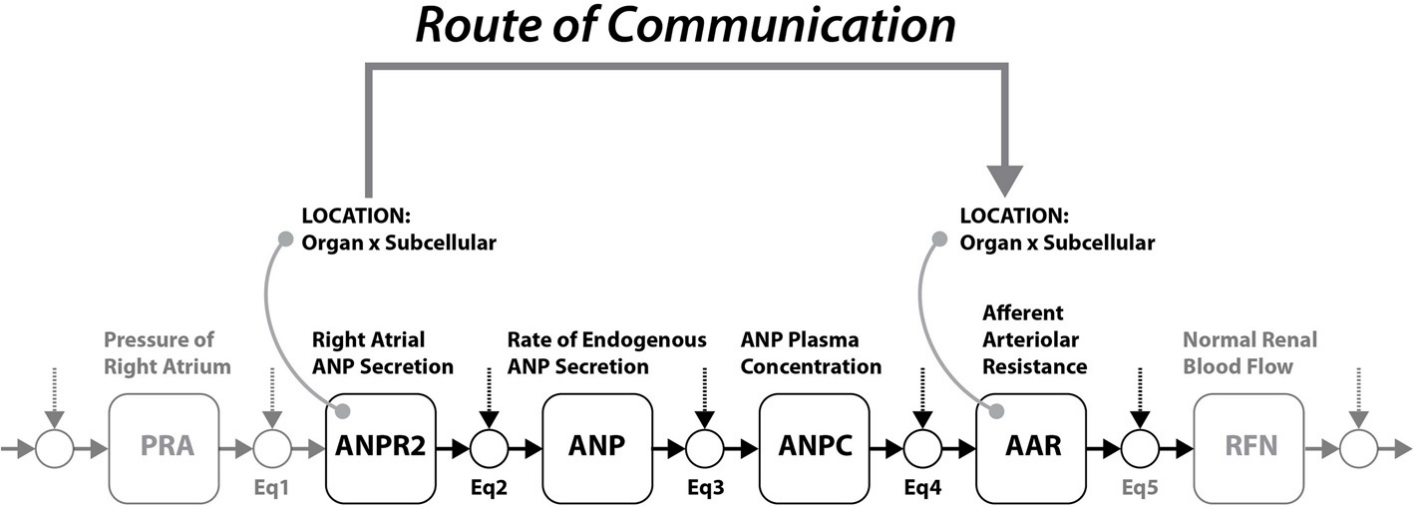
**State variables in the Guyton model.** llustration of six state/rate variables interlinked by a network of five ordinary differential equations (Eq1‐5) from the Guyton model of circulation [12]. Note that the variable labelled ANPR2 bears no relation to the ANP receptor (ANPr).

In addition, this method is also relevant for the integration of mathematical models of physiological processes. For instance, Figure 5 shows six variables from the Guyton model of circulation [12]. From the annotation of these variables with ontologies including, for example, FMA and GO, the location knowledge associated with variables ANPR2 and AAR provides the information necessary to calculate the route that conveys the quantified influence of the biological process depicted by variable ANPR2 onto the site of ANP interaction with its receptor that is implicit in variable AAR. As this route is defined in terms of anatomical and subcellular sites, the result of this calculation may provide a way to discover novel interactions between these two variables and other similarly annotated Guyton variables. By extension, the calculation of routes of communication can also support the functional linkage of variables from different models that have been annotated in a similar manner. For instance, this approach may support the integration of the Guyton model with a detailed pharmacodynamic model of ANP signalling that takes ANP receptor occupancy as its independent variable.

## Conclusion

The work presented in this paper aims to support research in intercellular communication by enabling knowledge‐based inference on physiologically‐related biomedical data and models. The prospect of automating such a process is greatly enhanced by the use of widely‐supported biomedical ontologies for multiscale anatomy in the annotation of data and models. The methodology presented contributes key groundwork for the communal curation and visualisation of physiology pathways. From a practical drug discovery and development perspective, our methodology also provides an approach to study of the influence that diseases interfering with transport processes may have on the administration, absorption, distribution and excretion routes of a drug. The proposed methodology will benefit from ongoing efforts to extend its knowledge representation basis and improve the integration of data and models via the application of multiscale biomedical knowledge.

## Competing interests

Both authors declare that they have no competing interests.

## Authors’ contributions

The work and the redaction of the paper is the result of the authors’ collaboration. BdB curated the knowledge of connectivity for the cardiovascular system and produced the corresponding graph. PG formalised the theory presented and developed the prototype implementation. The authors designed the illustrative scenario together based on BdB’s expertise in physiology and PG’s expertise in knowledge representation. PG produced a first draft of the paper which was subsequently collaboratively worked on and agreed upon. Both authors read and approved the final manuscript.
